# (−)-Epigallocatechin-3-gallate (EGCG) attenuates salt-induced hypertension and renal injury in Dahl salt-sensitive rats

**DOI:** 10.1038/s41598-020-61794-6

**Published:** 2020-03-16

**Authors:** Dan Luo, Jianping Xu, Xuejiao Chen, Xu Zhu, Shuang Liu, Jie Li, Xinting Xu, Xiao Ma, Jinhua Zhao, Xu Ji

**Affiliations:** 1 Key Laboratory of Medicinal Chemistry for Natural Resource, Ministry of Education and Yunnan Province, School of Chemical Science and Technology, Yunnan University, Kunming, Yunnan 650091 China; 2 State Key Laboratory of Phytochemistry and Plant Resources in West China, Kunming Institute of Botany, Chinese Academy of Sciences, Kunming, Yunnan 650201 China; 3 Department of Respiratory and Critical Disease, Xi’an International Medical Center Hospital, Xi’an, Shaanxi 710100 China; 4 Key Laboratory of Pu-erh Tea Science of Ministry of Education, Yunnan Research Center for Advanced Tea Processing, Yunnan Agricultural University, Kunming, China, No. 452 FengYuan Street, Kunming, 650201 Yunnan China

**Keywords:** End-stage renal disease, Renal fibrosis

## Abstract

Epigallocatechin-3-gallate (EGCG), a main active catechin in green tea, was reported to attenuate renal injury and hypertension. However, its effects on salt-induced hypertension and renal injury remain unclear. In the present study, we explored its effects on hypertension and renal damage in Dahl rats with salt-sensitive hypertension. We found that EGCG could lower blood pressure after 6 weeks of oral administration, reduce 24 h urine protein levels and decrease creatinine clearance, and attenuate renal fibrosis, indicating that it could attenuate hypertension by protecting against renal damage. Furthermore, we studied the renal protective mechanisms of EGCG, revealing that it could lower malondialdehyde levels, reduce the numbers of infiltrated macrophages and T cells, and induce the apoptosis of NRK-49F cells. Considering that the 67 kD laminin receptor (67LR) binds to EGCG, its role in EGCG-induced fibroblast apoptosis was also investigated. The results showed that an anti-67LR antibody partially abrogated the apoptosis-inducing effects of EGCG on NRK-49F cells. In summary, EGCG may attenuate renal damage and salt-sensitive hypertension via exerting anti-oxidant, anti-inflammatory, and apoptosis-inducing effects on fibroblasts; the last effect is partially mediated by 67LR, suggesting that EGCG represents a potential strategy for treating salt-sensitive hypertension.

## Introduction

Salt-sensitive hypertension is characterized by a significant increase in blood pressure after high salt intake. Patients with this condition are at high risk for cardiovascular and renal morbidity and mortality. Renal injury plays a key role in the pathogenesis of salt sensitive hypertension^1^. Recently, the roles of oxidative stress and inflammatory cell infiltration in the kidney in the pathogenesis of salt-sensitive hypertension were verified^2,3^. Renal fibrosis is a common pathway for a variety of chronic kidney diseases to progress to end-stage renal failure^4^ and excessive proliferation of renal interstitial fibroblasts participates in the development of renal fibrosis^5,6^.

(−)-Epigallocatechin-3-gallate (EGCG) is the most active and abundant polyphenol in green tea, accounting for approximately 50% of green tea polyphenols^7^. Recently, EGCG has attracted interest due to its wide range of biological activities, such as its antioxidant, NO-scavenging, anti-inflammatory, anti-arthritic and apoptosis-inducing effects in multiple types of tumour cells^8,9^. EGCG benefits a broad spectrum of hypertension disorders, including renovascular hypertension^10^ and spontaneous hypertension^11^. The renoprotective effect of EGCG has been reported in several renal disease models^12^, such as acute kidney injury^13^, cisplatin-induced nephrotoxicity^14^, obstructive nephropathy^15^, glomerulonephritis^16^, lupus nephritis^17^, diabetic nephropathy^18^, and high-fat diet-induced kidney injury^19^. However, the effects of EGCG on salt-induced hypertension and renal injury remain unclear.

The non-integrin cell surface receptor, 67-kDa laminin receptor (67LR) is highly expressed on the surfaces of various tumour cells and is widely recognized as a molecular marker of metastatic aggressiveness^20,21^. Moreover, 67LR is a membrane receptor of EGCG^22^, and the binding of EGCG to the 67LR protein reportedly mediates many of EGCG’s beneficial activities, such as its anti-proliferative and apoptosis-inducing effects on multiple tumour cells^23^, antioxidant effects^24,25^, and anti-inflammatory activities^26,27^.

In this study, the effects of EGCG on hypertension and renal damage in Dahl salt-sensitive (Dahl/SS) rats were investigated, and the antihypertensive and renoprotective mechanisms of EGCG were also investigated. Additionally, to explore the role of 67LR in the renoprotection of EGCG, the role of 67LR in the apoptosis-inducing effects of EGCG on renal interstitial fibroblasts from rats was studied *in vitro*.

## Results

### Effects of EGCG treatment on body weight, food intake, heart rate and blood pressure

**Figure 1:**
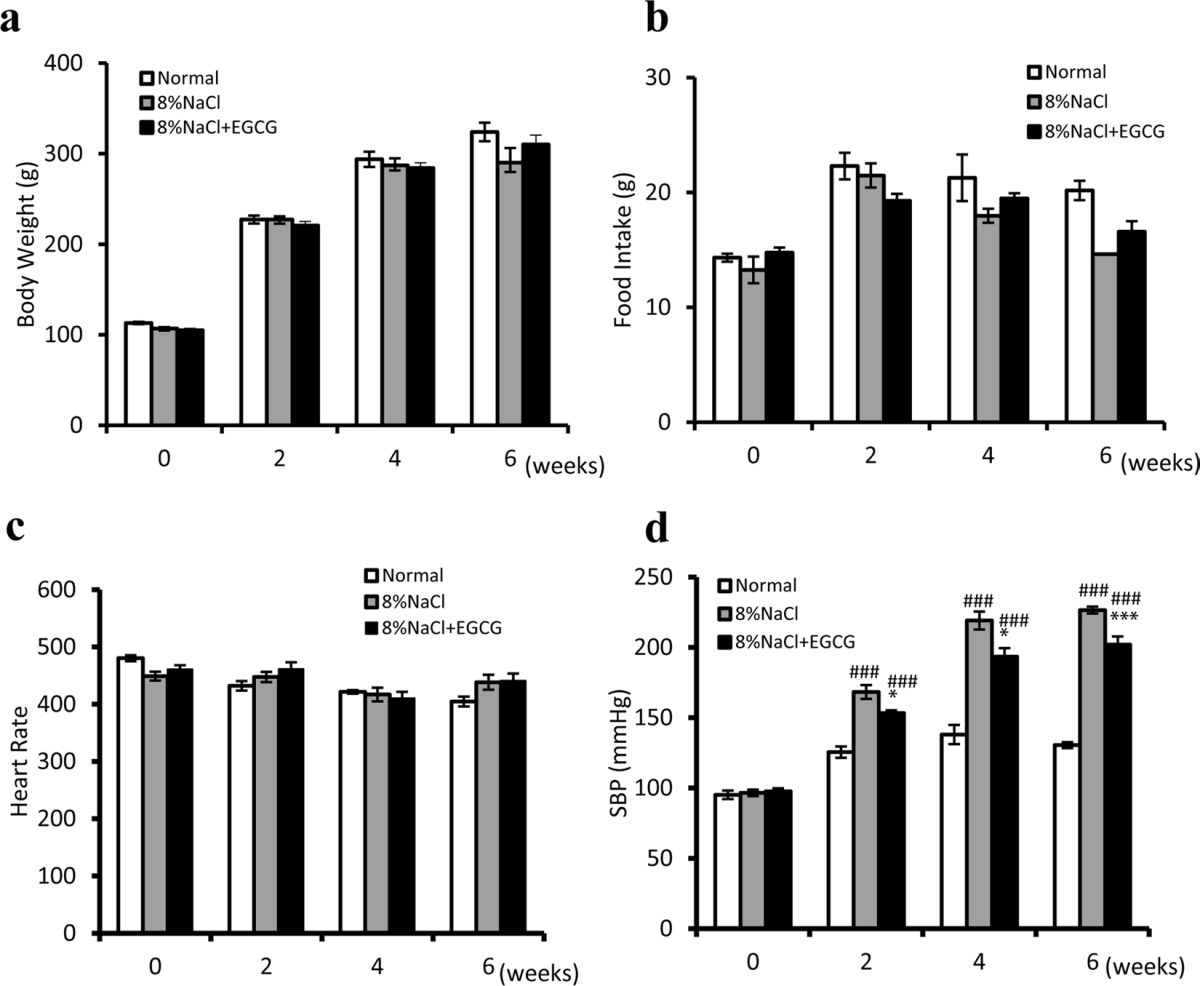
Effects of EGCG treatment on body weight (**a**), food intake (**b**), heart rate (**c**), and blood pressure (**d**) in Dahl/SS rat kidneys. Animals in the control group was administered the normal diet for 6 weeks. The rats in the model group were administered only the high-salt diet for 6 weeks. The rats in the EGCG group were fed a high-salt diet and orally administered EGCG. ^###^*P* < 0.001, compared with the control group; ^*^P < 0.05, ^***^P < 0.001, compared with the model group. n = 8.

As shown in Fig. 1a, rats showed no significant differences in body weight among the normal, model and EGCG groups. Although animals in the model and EGCG groups had lower food intake than those in the normal group after six weeks, there were no significant differences in food intake between the model and EGCG group animals(Fig. 1b). As shown in Fig. 1c, EGCG treatment did not affect heart rate, as measured by the tail-cuff method. Systolic blood pressure (SBP) significantly increased after consumption of an 8% NaCl diet (*P* < 0.001), and EGCG treatment significantly attenuated this increase in SBP (Fig. 1d. at week 6, *P* < 0.001).

### Effects of EGCG treatment on renal function and renal fibrosis in Dahl/SS rats

**Figure 2:**
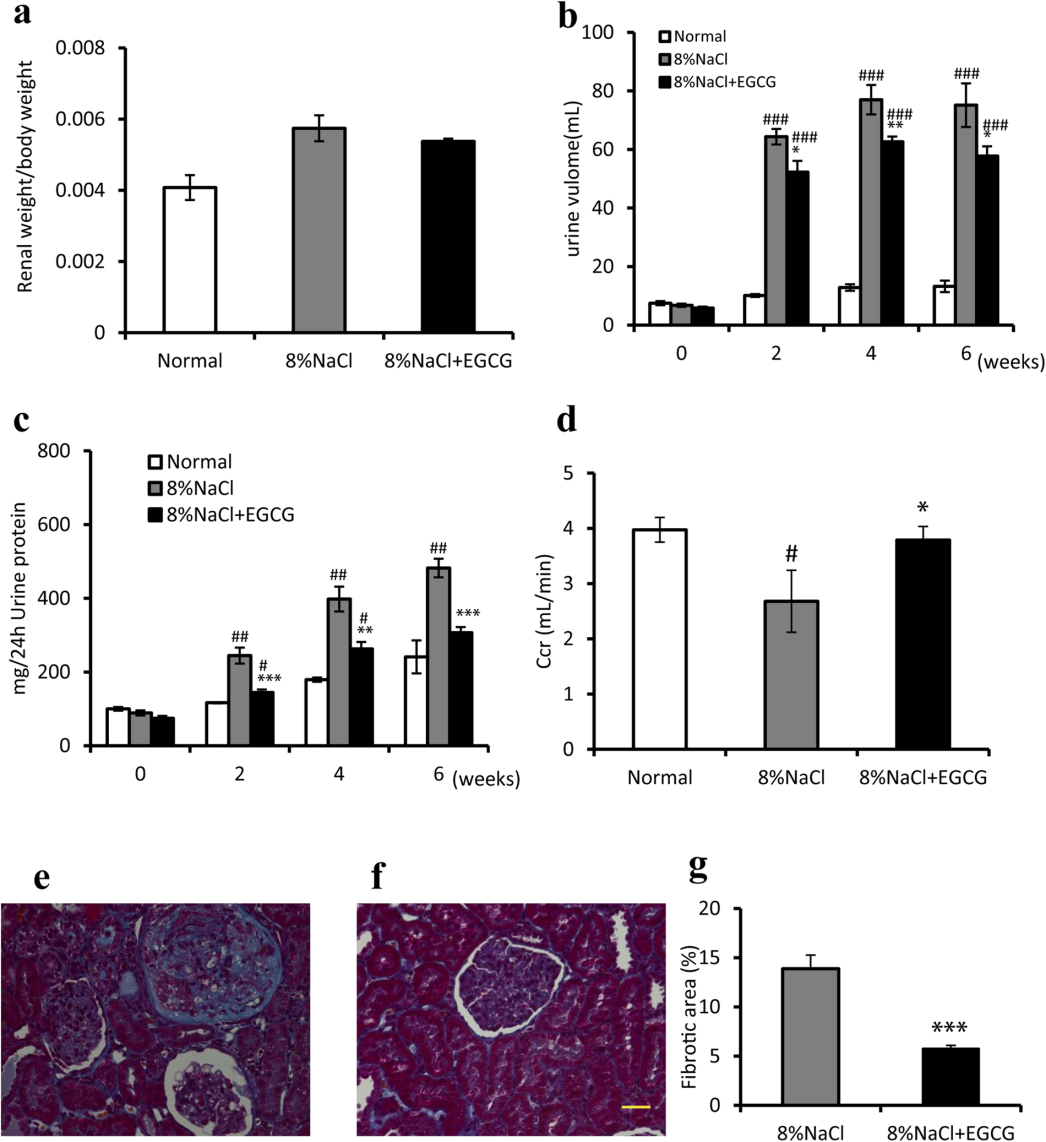
Effects of EGCG treatment on renal weight (**a**), urinary volume (**b**), urine protein (**c**), creatinine clearance (C_Cr_) (**d**), and renal fibrosis (**e**–**g**). Kidneys were harvested at week 6, and renal organ weight per body weight was calculated (**a**). Urine volume (**b**) was collected, and 24 h urine protein (**c**) levels were determined every two weeks. Creatinine clearance (C_Cr_) was measured at 6 weeks, and calculated using the following formula: C_Cr_ = (U_Cr_ X V)/P_Cr_, where U_Cr_ is the concentration of urinary creatinine (mg·dL^−1^), P_Cr_ is the concentration of plasma creatinine (mg·dL^−1^), and V is the urine flow rate (mL·min^−1^). C_Cr_ was significantly increased after 6 weeks of a high-salt diet, and this increase was improved by 6 weeks of EGCG treatment (**d**). Representative images (400×) of kidneys in the model (**e**) and EGCG (**f**) groups stained with Masson’s trichrome revealing interstitial fibrosis and glomerular sclerosis (blue area). EGCG treatment markedly reduced the percentage of fibrotic areas (**g**). ^*#*^*P* < 0.05, ^#*#*^*P* < 0.01, ^###^*P* < 0.001, compared with the control group (normal diet); ^*^P < 0.05, ^**^P < 0.01, ^***^P < 0.001, compared with the model group (8% NaCl). n = 8. Scale bars = 50 μm.

There was no significant difference in renal weight between the model and EGCG groups at week 6 (Fig. 2a). After being fed an 8% NaCl diet feeding, animals in both the model and EGCG groups exhibited increased urine volume compared with that of control group animals. Furthermore, EGCG could significantly reduce the urine volume in the model group (Fig. 2b. at week 6, *P* < 0.05), which indicated that EGCG treatment may reduce renal function damage. Other results also showed that urinary protein excretion was significantly attenuated after EGCG treatment (Fig. 2c. at week 6, model group *vs*. EGCG group; 13.88 ± 1.38% vs 5.76 ± 0.32%; *P* < 0.001), and creatinine clearance (C_Cr_) was considerably improved after 6 weeks of EGCG treatment (Fig. 2d, *P* < 0.05).

Six weeks of an 8% NaCl diet caused tubular dilatation, interstitial fibrosis, and glomerular sclerosis (Fig. 2e); but EGCG treatment significantly attenuated these pathological changes (Fig. 2f) and markedly reduced fibrotic areas (Fig. 2g, *P* < 0.001).

### Effects of EGCG treatment on renal oxidative stress in Dahl/SS rats

**Figure 3:**
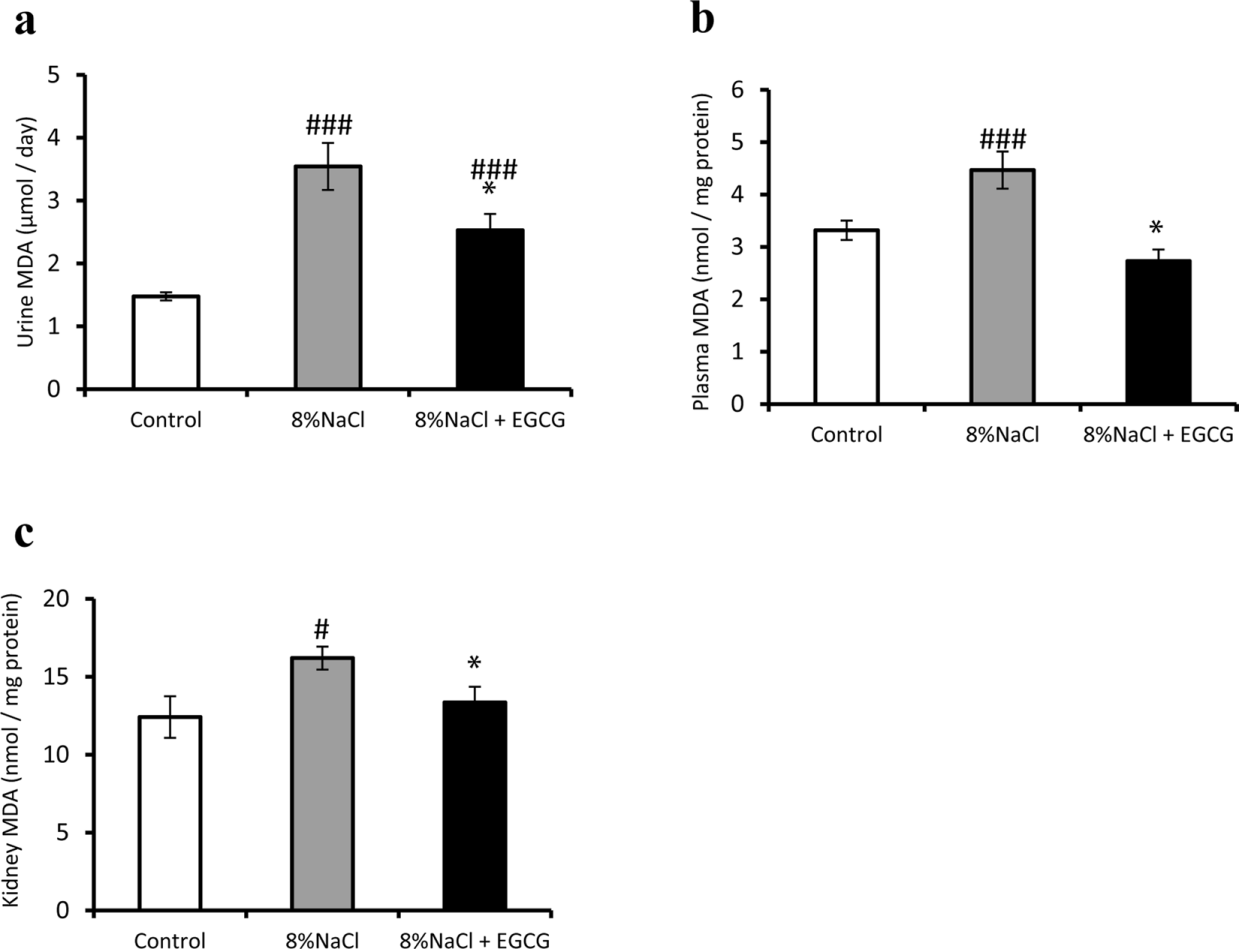
Effects of EGCG treatment on renal oxidative stress. MDA (a marker of oxidative stress) was measured using the TBARS assay kit. Compared with the normal control, high-salt treatment significantly increased oxidative stress, and EGCG treatment significantly attenuated this increase in the urine (**a**), serum (**b**), and kidney (**c**). ^*#*^*P* < 0.05, ^###^*P* < 0.001, compared with the control group (normal diet); ^*^P < 0.05, compared with the model group (8% NaCl). n = 8.

Lipid peroxidation is a well-established mechanism of cellular injury, and malondialdehyde (MDA) is a naturally occurring product of lipid peroxidation^28^. In this study, MDA was used as an indicator of oxidative stress in cells and tissues. The MDA levels in the urine, serum and kidney were measured using a commercially available TBARS assay kit and according to the manufacturer’s instructions. As shown in Fig. 3, high salt treatment significantly increased oxidative stress in the urine (Fig. 3a, *P* < 0.001), plasma (Fig. 3b, *P* < 0.001) and kidney (Fig. 3c, *P* < 0.05), and this increase was significantly attenuated after EGCG treatment (Fig. 3a–c, *P* < 0.05).

### Effects of EGCG treatment on the renal infiltration of immune cells in Dahl/SS rats

**Figure 4:**
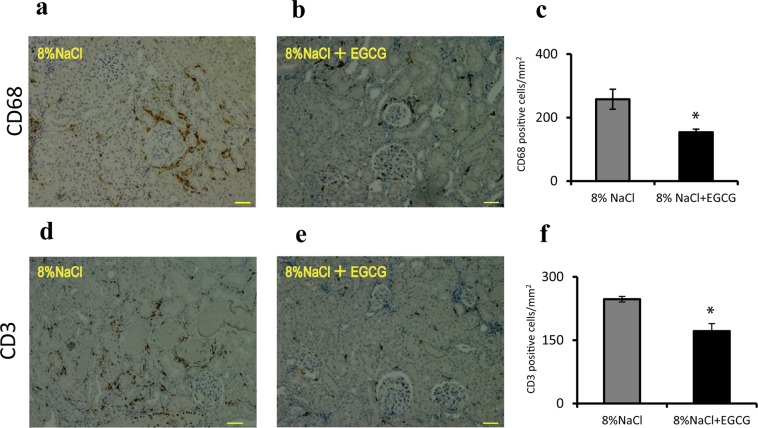
Effects of EGCG treatment on immune cell infiltration in Dahl/SS rat kidneys. Immunohistochemical assays revealed macrophages (**a**,**b**) and T cells (**c**,**d**) (brown area), which were identified by positive staining with anti-CD68 and anti-CD3 antibodies, respectively. The microscopic findings (400×) showed that macrophage and T-cell infiltration in the EGCG group was markedly decreased compared with that in 8% NaCl goup. The numbers of cells positive for CD68 and CD3 were counted and compared between the model group (8% NaCl) and EGCG group (8% NaCl + EGCG). EGCG treatment markedly attenuated macrophage (**c**) and T-cell (**f**) infiltration. ^*^P < 0.05, compared with the model group. n = 8. Scale bars = 50 μm.

To evaluate the effects of EGCG on renal inflammation, kidney tissue sections were stained with CD68 (a macrophage marker) or CD3 (a T-cell marker). As shown in Fig. 4, significant differences were observed between the high-salt and EGCG groups with respect to macrophage (Fig. 4a–c*, P* < 0.05) and T-cell infiltration (Fig. 4d–f, *P* < 0.05), suggesting that EGCG could effectively attenuate renal inflammation in rats with salt-sensitive hypertension.

### Effects of EGCG treatment on renal interstitial fibroblasts

**Figure 5:**
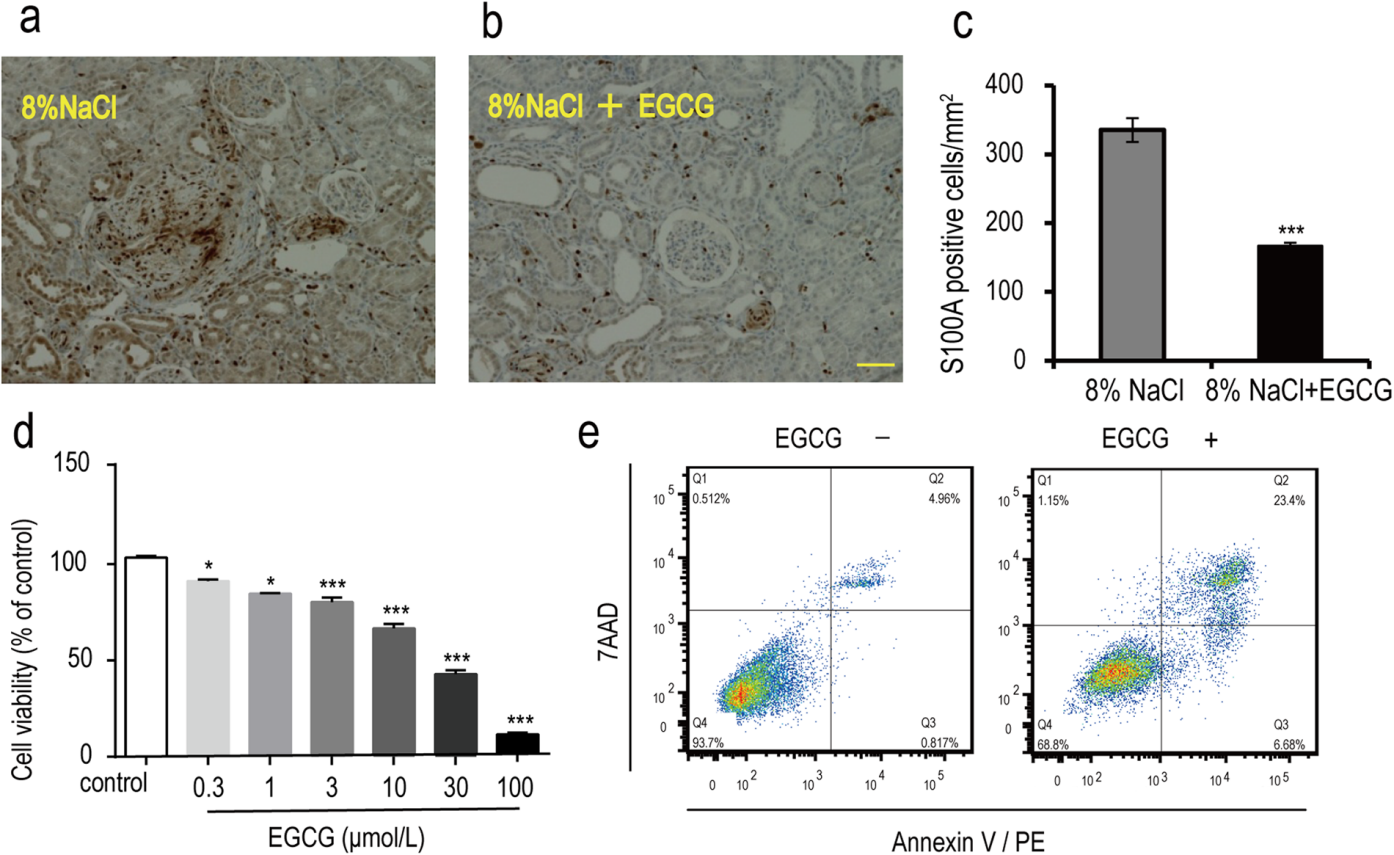
The effect of EGCG on renal interstitial fibroblasts. Immunohistochemical examination revealed fibroblasts (brown area), which were identified by positive staining with an anti-S100A4 (a specific fibroblast marker) antibody. Representative photographs of immunohistochemical staining (400×) showed that EGCG treatment (**a**) reduced the number of S100A-positive cells in the kidney compared with that in the model group (**b**). The average numbers of S100A-positive cells per mm^2^ are also shown (**c**). ^***^P < 0.001, compared with the model group, n = 8. Scale bars = 50 μm. To confirm that EGCG could directly inhibit the proliferation of renal fibroblasts, its effects on cultured renal interstitial fibroblasts of rats (NRK-49F cells) were evaluated. NRK-49F cells were treated with EGCG (0.3 μmol/L ~ 100 μmol/L) for 48 h, and the cell viability was then measured by the MTS assay (**d**). ^*^P < 0.05, ^***^P < 0.001, compared with control. n = 5. Then, to evaluate the effect of EGCG (20 μmol/L) on the apoptosis of NRK-49F cells, apoptotic cells were stained with annexin V/PE and 7AAD and analysed by flow cytometry (**e**).

S100A4 is a specific fibroblast marker. As shown in Fig. 5, compared with that of the normal group, the number of S100A4-positive cells in the fibrotic areas of the EGCG treatment group was significantly decreased (Fig. 5c, *P* < 0.001), indicating that EGCG could inhibit renal fibroblast proliferation (Fig. 5a–c). To confirm that EGCG directly inhibits the proliferation of renal fibroblasts in salt-sensitive rats, its effects on cultured renal interstitial fibroblasts harvested from rats (NRK-49F cells) were evaluated at the cell level. As shown in Fig. 5d, EGCG (0.3 ~ 100 μmol/L) reduced the cell viability of NRK-49F cells in a dose-dependent manner. To ascertain whether EGCG could induce the apoptosis of NRK-49F cells, the cells were stained with annexin V/PE and 7AAD and analysed by flow cytometry. The results showed that EGCG (20 μmol/L) could induce the apoptosis of NRK-49F cells (Fig. 5e).

### The role of 67LR in the EGCG-induced apoptotic effects on NRK-49F cells

To examine whether the EGCG-induced apoptosis effects on NRK-49F cells were mediated by 67LR, the cells were pretreated with an anti-67LR antibody (MLuC5) or normal mouse IgM.

**Figure 6:**
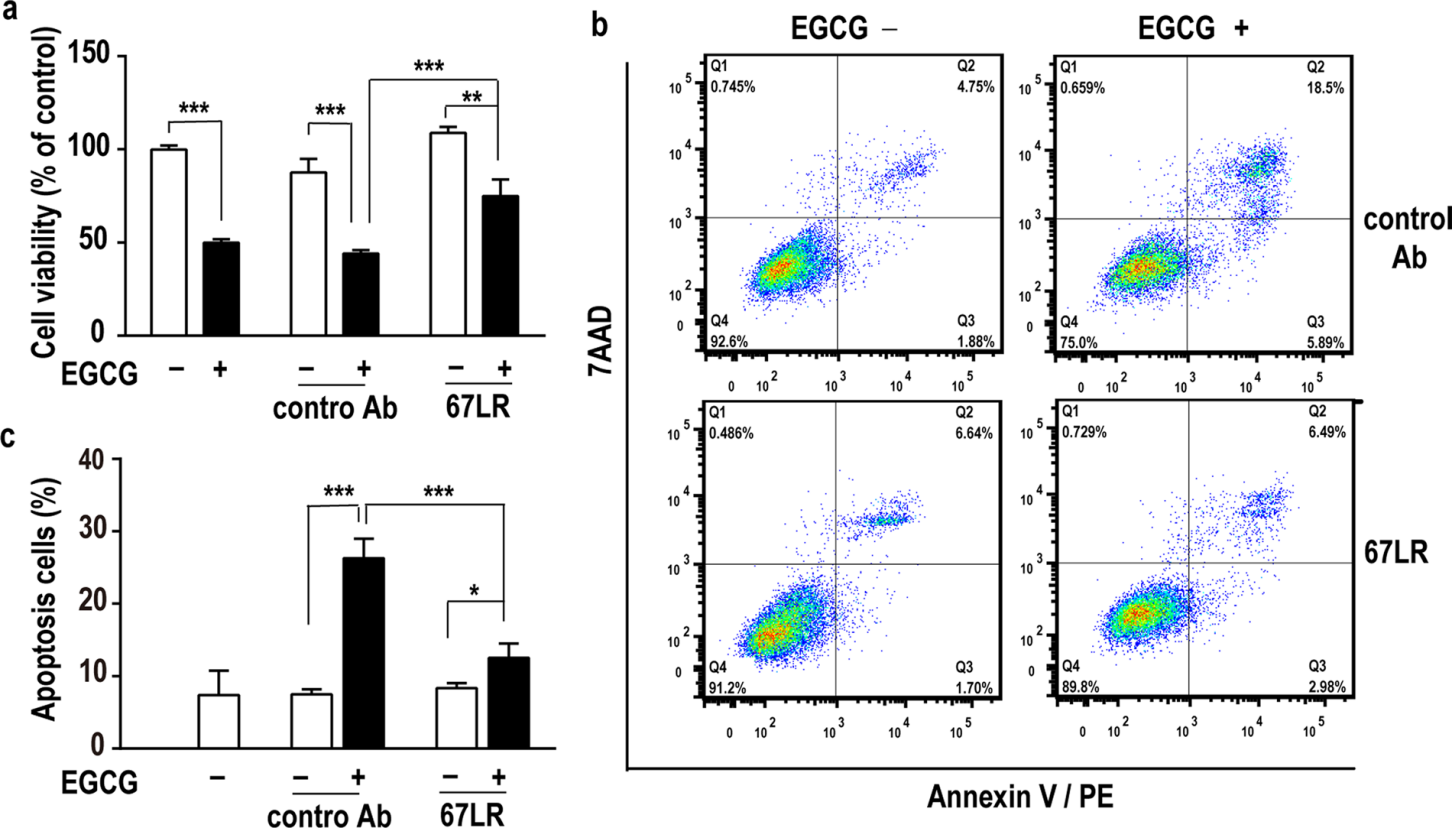
The role of 67LR in the apoptosis-induced effects of EGCG on cultured renal interstitial fibroblasts. NRK49F cells were preincubated for 1 h with an anti-67LR antibody or an IgM control antibody and then treated with or without 20 μmol/L EGCG for 48 hours. Cell viability was measured by the MTS assay (**a**). Moreover, apoptotic cells were stained with annexin V/PE and 7AAD and analysed by flow cytometry (**b**-**c**). ^*^P < 0.05, ^**^P < 0.01, ^***^P < 0.001, n = 3.

As shown in Fig. 6, EGCG (20 μmol/L) significantly reduced the cell viability of NRK-49F cells (*P* < 0.001), but after pretreatment with the anti-67LR antibody (20 μg/mL), the reduction in cell viability induced by EGCG was partially offset (Fig. 6a). Similarly, the cells pretreated with the anti-67LR antibody were partially protected from apoptosis induced by EGCG (Fig. 6b,c), suggesting that 67LR partially mediated the apoptosis-inducing effects of EGCG. Based on the above results, 67LR might play a role in the renoprotective effects of EGCG.

### Effects of EGCG treatment on renal 67LR expression in Dahl/SS rats

To explore the role of 67LR in the renoprotective effects of EGCG, the distribution of 67LR protein expression in the kidney and the effects of EGCG on 67LR expression were further studied. According to our study, immunostaining of 67LR in the kidney showed expression in the epithelial cells of proximal tubules, epithelial cells of renal capsules, podocytes, vascular endothelial cells, and fibroblasts, indicating that 67LR may play a certain physiological role in the kidney. Furthermore, to evaluate the effects of EGCG treatment on renal 67LR expression, we investigated 67LR expression by RT-PCR, Western blot, and immunofluorescence.

**Figure 7:**
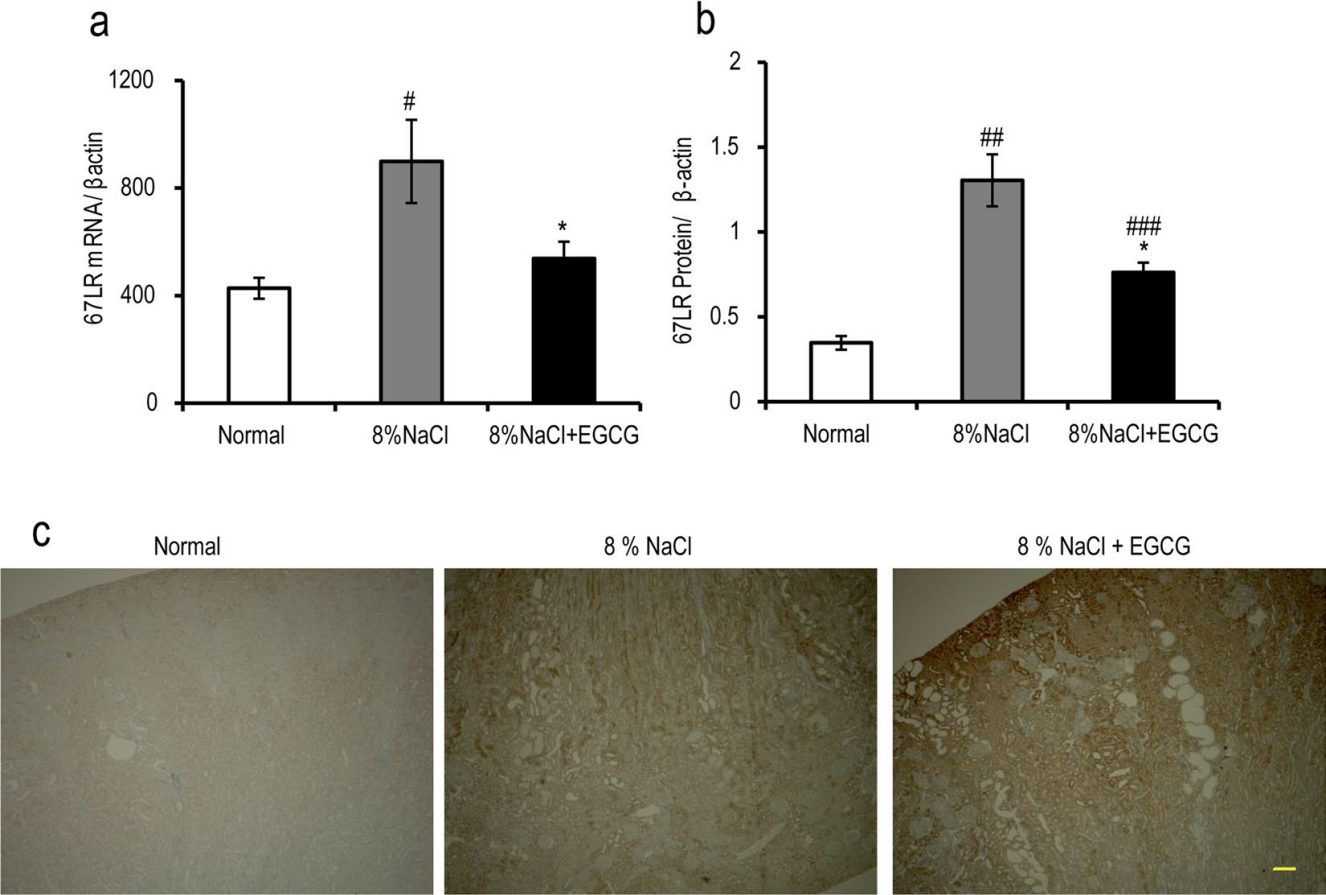
67LR expression in Dahl/SS rat kidneys. Analyses of 67LR mRNA (**a**) and 67LR protein (**b**) expression in the kidneys showed that the mRNA and protein expression levels of 67LR in the kidneys were increased in rats of the 8% NaCl diet and 8% NaCl diet plus EGCG groups, compared with those of rats in the normal group. EGCG treatment sharply decreased this increase in the model group. Representative photographs of immunohistochemical staining (100×) showed that compared with the control group, the expression of 67LR in the kidney was increased in the model group, but compared with the model group, EGCG treatment downregulated 67LR levels. ^*#*^*P* < 0.05, ^#*#*^*P* < 0.01, ^#*#*#^*P* < 0.001, compared with the control group (normal diet); ^*^P < 0.05, compared with the model group (8% NaCl), n = 8. Scale bars = 50 μm.

As shown in Fig. 7, the expression of 67LR was increased at the mRNA and protein levels after high-salt diet treatment. 67LR mRNA expression in the kidney decreased sharply in the EGCG-treated rats compared with the high-salt rats (Fig. 7a, *P* < 0.05). Analysis of 67LR expression by Western blot revealed the same dramatic decrease in the kidney after EGCG treatment (Fig. 7b). Immunostaining of renal 67LR showed the same trend (Fig. 7c).

## Discussion

EGCG has beneficial effects on a broad spectrum of hypertension disorders, including renovascular hypertension^10^ and spontaneous hypertension^11^. EGCG has been reported to protect renal function in several renal disease models^12^, such as acute kidney injury^13^, cisplatin-induced nephrotoxicity^14^, and obstructive nephropathy^15^. However, the effects of EGCG on salt-sensitive hypertension remain unclear. In this study, we observed that EGCG treatment could decrease SBP, reduce proteinuria, and ameliorate renal fibrosis in Dahl rats with salt-sensitive hypertension and attenuate salt-induced hypertension via renoprotective effects. Further studies revealed that the antihypertensive and renoprotective effects of EGCG could be attributed to its antioxidant, anti-inflammatory, and apoptosis-inducing effects. Furthermore, 67LR might partially mediate the apoptosis-inducing effects of EGCG. Our work demonstrated the antihypertensive effects of EGCG on rats with salt-sensitive hypertension and the potential mechanisms. Moreover, it revealed a possible therapeutic target of EGCG and a possible pathomechanism underlying salt-induced kidney damage; however, these results require further research.

Dahl salt sensitive rats are widely used to investigate salt-sensitive hypertension, which is characterized by a significant increase in blood pressure after high salt intake. Herein, the rat blood levels increased after 6 weeks of 8% NaCl intake, a result that is consistent with those of previous studies^29,30^, suggesting that the salt-sensitive hypertension model was successfully duplicated.

Although multiple factors participate in controlling arterial blood pressure, the kidney is a pivotal factor. It regulates salt and water excretion and controls peripheral vascular tone to regulate arterial blood pressure. Renal injury is one of the pathomechanisms of salt sensitive hypertension. The study of kidney cross-transplantation between normotensive and salt-sensitive hypertensive rats confirmed the central role of the kidney in salt-sensitive hypertension^31^. In this study, EGCG improved renal function and attenuated renal fibrosis, which indicates that it may lower blood pressure via exerting renal protective effects.

The kidney is susceptible to oxidative stress and inflammation after exposure to harmful agents^32^. Recently, the roles of oxidative stress and renal inflammatory cell infiltration in the development of salt-sensitive hypertension were verified^33,34^. After antioxidant treatment or specific NADPH oxidase knockout, the level of oxidative stress in the kidney was reduced, and salt-sensitive hypertension and the related renal injury were improved^33,35^. The present study demonstrated that EGCG could attenuate oxidative stress and infiltration of immune cells, suggesting that EGCG could attenuate salt-sensitive hypertension and renal damage via antioxidant and anti-inflammatory effects. Similarly, previous studies^10–12^ also demonstrated that EGCG could attenuate renal damage or hypertension via antioxidant and anti-inflammatory effects. Taken together, these studies revealed the common pharmacological characteristics of EGCG.

Additionally, due to the important role of renal interstitial fibroblasts in the development of renal fibrosis^5,6^, we investigated the effects of EGCG on renal interstitial fibroblasts *in vivo* and *in vitro*. The results showed that EGCG protected the kidneys from injury by reducing the number of renal interstitial fibroblasts, and the reduction in fibroblast numbers was potentially attributed to the apoptosis-inducing effects of EGCG.

67LR is a membrane receptor of EGCG^22^ and EGCG was reported to trigger cell death via 67LR in multiple types of tumour cells^23,36^. Previous studies showed that EGCG could activate the 67LR signalling pathway to inhibit inflammation in endothelial cells^27^, adipocytes^26^ and intestinal epithelial cells^37^. EGCG remarkably decreased oxidative stress and inflammation levels through 67LR in an acute lung injury mouse model^24^, and EGCG inhibited H_2_O_2_-induced apoptosis via 67LR in mouse vascular smooth muscle cells, indicating that the antioxidant effect of EGCG is associated with 67LR^25^. Therefore, we explored the role of 67LR in the apoptosis-inducing effects of EGCG on NRK-49F cells. We found that 67LR might have partially mediated the apoptosis-inducing effects of EGCG. In addition, EGCG effectively attenuated renal oxidative stress and inflammation. Therefore, we speculated that EGCG might protect the kidney from oxidative stress and inflammation through the 67LR-signalling pathway. The role of 67LR in the antihypertensive and renoprotective effects of EGCG should be further verified *in vivo*.

Although 67LR has been well studied in various tumour cells^20,21^, few studies on its expression in the kidney have been conducted. In this study, we found that 67LR was expressed in the epithelial cells of the proximal tubules, the epithelial cells of the renal capsules, podocytes, vascular endothelial cells, and fibroblasts in the kidney. We also observed that high-salt treatment increased renal 67LR mRNA and protein levels, compared with those of the control group. These novel findings indicated that 67LR might play a certain physiological role and be a marker of kidney injury.

In general, upon receptor agonization, the expression level of the receptor should not be altered. Our results showed that EGCG decreased renal 67LR mRNA and protein levels. The reduction in 67LR expression might have been due to the role of 67LR in the pathology of renal injury. 67LR is reported to be a molecular marker of metastatic aggressiveness^20,21^. Renal epithelial cells could be transformed to the mesenchymal cell phenotype and acquire the ability to migrate and invade, which could promote the development of renal fibrosis^38^. We speculated that upregulation of 67LR expression might be one of the pathomechanisms of renal injury and that EGCG can protect against renal damage by reducing 67LR expression. However, the role of 67LR in the progression of renal injury needs to be further investigated in salt-sensitive and salt-resistant rats.

In this study, EGCG was administered at a dose of 50 mg/kg/12 h. If this dose were converted to a human dose based on the body surface area calculation, a person with a weight of 70 kg weight would take 1120 mg of EGCG per day. According to the previous study^39^, a cup of green tea (250 mL) made from 2.5 g of tea leaves contains approximately 240 ~ 320 mg of catechins, and the content of EGCG in catechins is approximately 60 ~ 65%^40^. Given the EGCG content in green tea, it can be estimated that for a person of 70 kg, five to seven cups of green tea per day would provide a sufficient dose of EGCG. More research needs be conducted to provide further valuable evidence to guide the clinical use of green tea in the prevention and treatment of salt-induced renal injury and hypertension.

In conclusion, EGCG may attenuate salt-sensitive hypertension and renal damage by exerting antioxidant, anti-inflammatory and apoptosis-inducing effects which might be partly mediated by 67LR. This finding may lead to not only a better understanding of the biological roles of EGCG, but also to potential clinical applications for salt-sensitive hypertension.

## Methods

### Experimental animals

Dahl salt-sensitive (Dahl/SS) rats were purchased from Beijing Vital River Laboratory Animal Technology Co., Ltd. (Beijing, China), fed a normal or high-salt diet (0.5% or 8% NaCl, respectively) in a temperature-controlled and pathogen-free room (no. SYXK K2013-0004), and provided tap water ad libitum. Experiments were conducted according to the guidelines of the National Institutes of Health Guide for the Care and Use of Laboratory Animals. All experimental protocols were pre-approved by the Experimental Animal Ethics Committee of Kunming Institute of Botany, Chinese Academy of Sciences.

### Renoprotective effects of EGCG in Dahl/SS rats

EGCG that was more than 95% pure (Sigma-Aldrich, E4143, USA) was used in the following studies. Four-week-old male Dahl/SS rats (100 ~ 110 g) were divided into three groups: control (0.5% NaCl, n = 8), model (8% NaCl, n = 8), and EGCG (8% NaCl + EGCG, n = 8). The composition of the normal diet is shown online in Supplementary Table S1. The high salt diet differed from the normal diet in regard to its NaCl content. The rats in the EGCG group were orally administered EGCG (50 mg/kg body weight) twice daily for 6 weeks. Blood pressure was measured every 2 weeks using a tail-cuff method (BP-2010A; Softron, Beijing, China). The 24 h urine samples were collected from rats housed in metabolic cages every 2 weeks.

Urinary protein levels were measured using a BCA protein assay kit (Thermo Scientific, Rockford, IL, USA). C_Cr_ at week 6 was calculated as described previously^41,42^, and creatinine levels were measured using a creatinine assay kit (DICT-500; BioAssay Systems, Hayward, CA, USA).

### Measurement of 67LR mRNA in the kidneys of Dahl/SS rats

After 6 weeks with or without EGCG treatment, the rats were anaesthetized with pentobarbital (25 mg·kg^−1^). According to a previous method^42^, plasma and serum were prepared, and the left kidneys of the rats were removed, weighed, and sectioned longitudinally. One half of the kidney was frozen in liquid nitrogen and deep-frozen at −80 °C before measurement. The other half was used to isolate RNA with TRIzol reagent (Invitrogen, Carlsbad, CA, USA) according to the manufacturer’s instructions, and the expression level of 67LR mRNA was determined by real-time RT-PCR using a commercial kit (Applied Biosystems, Foster City, CA, USA) and normalized to that of β-actin^41^. These measurements are expressed as log_10_ (2^35-CT1^/2^25-CT2^), where 2^35-CT1^ and 2^25-CT2^ correspond to the expression levels of 67LR and βactin mRNA, respectively, as described previously^41^.

### Western blotting

Frozen kidney tissues were homogenized in 1% NP-40 lysis buffer (Beyotime Biotechnology, Shanghai, China). Forty micrograms of protein was separated by SDS-PAGE (10% gels), transferred to a PVDF membrane (0.45 µm; Merck Millipore, USA), blocked with 5% (w/v) skim milk powder, and incubated overnight at 4 °C with primary antibodies diluted 1:500. Membrane-bound antibodies were detected with a horseradish peroxidase-conjugated secondary antibody (Santa Cruz Biotechnology, USA) diluted at 1:10000 and visualized by an ECL advanced Western blotting detection kit (GE Healthcare, USA). β-Actin (Santa Cruz Biotechnology, USA) served as a loading control.

### Histological examination

As previously reported^41,43^, after high-salt intake with or without EGCG treatment for 6 weeks, the right kidney of the rats were dissected, fixed in 10% formalin overnight at 4 °C and subsequently embedded in paraffin. Five-micromet-thick kidney sections were stained with Masson’s trichrome. Ten high-power fields (400 × magnification) for each kidney were randomly captured with a fluorescence microscope (Ti-E, Nikon, Japan), and renal fibrosis was measured and analysed with NIS-Elements (Nikon, Tokyo, Japan) and MetaMorph software (Molecular Devices, Sunnyvale, CA).

### Immunohistochemistry

Immunohistochemistry was used to detect 67LR, macrophages (CD68), T cells (CD3), and fibroblasts (S100A4). After deparaffinization and antigen retrieval, kidney sections were treated with 3.3% H_2_O_2_ to block endogenous peroxidase activity, and blocked with 5% normal goat serum. The sections were incubated overnight at 4 °C with primary antibodies against 67LR (Abcam, Cambridge, UK), CD68 (Abcam), CD3 (BD Pharmingen, San Diego, CA, USA), and S100A4 (Abcam). The Simplestain MAX-PO (rat) kit (Nichirei, Tokyo, Japan) was used as a secondary antibody and allowed to incubate for 30 min at room temperature^42^. Bound antibody was visualized using 3,3'-diaminobenzidine (DAB; Beyotime Biotechnology, Shanghai, China), and nuclei were stained with haematoxylin. The images were captured with a fluorescence microscope (Nikon Ti-E) and the numbers of DAB-positive cells in the 10 random fields for each kidney were counted. The results are expressed as the number of positive cells per square millimetre of renal tissue.

### Measurement of MDA

The MDA levels in urine, plasma, and kidney samples, were measured with an enzyme-linked immunosorbent assay kit (Cayman 10009055, Michigan, USA).

### Cell viability assays

NRK-49F cells were purchased from the National Infrastructure of Cell Line Resource, and cultured in DMEM (Biology Industry, Israel) supplemented with 5% foetal bovine serum (FBS; Biology Industry) in a humidified incubator containing 95% air and 5% CO_2_ at 37 °C. To determine the effect of EGCG on NRK-49F cells, the cells were preincubated with 1% FBS, 200 units/ml catalase and 5 units/ml superoxide dismut ase (SOD; Sigma), and treated with EGCG at different concentrations (0.3~100 μmol/L) for 48 h. Cell viability was assayed using the CellTiter 96® AQueous One Solution Cell Proliferation Assay (MTS; Promega).

### Apoptosis detection

NRK-49F cells were cultured in 6-well plates and treated with EGCG (20 μmol/L) under the conditions described above. After 48 h of EGCG treatment, the cells were harvested, centrifuged and washed twice with cold PBS. Cell apoptosis was examined on a flow cytometer (FACS Celesta, Becton Dickinson, American) by Annexin V PE/7AAD kits (Becton Dickinson, American), according to the manufacturer’s instructions. The percentage of apoptotic cells was calculated using FlowJo. To examine the role of 67LR in the apoptotic effects of EGCG on NRK-49F cells, the cells were pretreated with an antibody(20 μg/mL) against 67LR (MLuC5) or normal mouse IgM for 1 h. The clone number of the anti-67LR antibody was the same as that in a previous study^22^.

### Statistical analysis

All data are expressed as the mean ± SEM. Statistical comparisons were performed by *t*-test for two groups, and by one-way ANOVA followed by Bonferroni’s test for more than two groups. *P* < 0.05 was regarded as a statistically significant difference.

## Supplementary information

Supplementary information
