# Author Correction: (−)-Epigallocatechin-3-gallate (EGCG) attenuates salt-induced hypertension and renal injury in Dahl salt-sensitive rats

**DOI:** 10.1038/s41598-020-67582-6

**Published:** 2020-06-29

**Authors:** Dan Luo, Jianping Xu, Xuejiao Chen, Xu Zhu, Shuang Liu, Jie Li, Xinting Xu, Xiao Ma, Jinhua Zhao, Xu Ji

**Affiliations:** 1 Key Laboratory of Medicinal Chemistry for Natural Resource, Ministry of Education and Yunnan Province, School of Chemical Science and Technology, Yunnan University, Kunming, 650091 Yunnan China; 2 State Key Laboratory of Phytochemistry and Plant Resources in West China, Kunming Institute of Botany, Chinese Academy of Sciences, Kunming, 650201 Yunnan China; 3 Department of Respiratory and Critical Disease, Xi’an International Medical Center Hospital, Xi’an, Shaanxi, 710100 China; 4 Key Laboratory of Pu-Erh Tea Science of Ministry of Education, Yunnan Research Center for Advanced Tea Processing, Yunnan Agricultural University, Kunming, China, No. 452 FengYuan Street, Kunming, 650201 Yunnan China

Correction to: *Scientific Reports* https://doi.org/10.1038/s41598-020-61794-6, published online 16 March 2020

The original version of this article contained errors. Xinting Xu was incorrectly affiliated with ‘Key Laboratory of Pu-erh Tea Science of Ministry of Education, Yunnan Research Center for Advanced Tea Processing, Yunnan Agricultural University, Kunming, China, No. 452 FengYuan Street, Kunming, 650201, Yunnan, China’. The correct affiliation is listed below:

Department of Respiratory and Critical Disease, Xi’an International Medical Center Hospital, Xi’an, Shaanxi, 710100, China

In addition, Xiao Ma was incorrectly affiliated with ‘Department of Respiratory and Critical Disease, Xi’an International Medical Center Hospital, Xi’an, Shaanxi, 710100, China’. The correct affiliation is listed below:

Key Laboratory of Pu-erh Tea Science of Ministry of Education, Yunnan Research Center for Advanced Tea Processing, Yunnan Agricultural University, Kunming, China, No. 452 FengYuan Street, Kunming, 650201, Yunnan, China

These errors have now been corrected in the PDF and HTML versions of the article and in the accompanying Supplementary Information file.

