# Supplementary material for: (−)-Epigallocatechin-3-gallate (EGCG) attenuates salt-induced hypertension and renal injury in Dahl salt-sensitive rats
Source: Sci Rep. 2020 Mar 16;10:4783. doi: 10.1038/s41598-020-61794-6 (PMC7075996; doi:10.1038/s41598-020-61794-6)
Supplement: Supplementary file 1 — Supplementary information [file 41598_2020_61794_MOESM1_ESM.docx]

**(**-**)-Epigallocatechin-3-gallate (EGCG) attenuates salt-induced hypertension and renal injury in Dahl salt-sensitive rats**

Dan Luo ^1,2,#^, Jianping Xu ^2,#^, Xuejiao Chen ^2^, Xu Zhu ^2^, Shuang Liu ^2^, Jie Li ^2^, Xinting Xu ^3^, Xiao Ma ^4,*^, Jinhua Zhao ^2,*^ , Xu Ji ^1,2,*^

^1^ Key Laboratory of Medicinal Chemistry for Natural Resource, Ministry of Education and Yunnan Province, School of Chemical Science and Technology, Yunnan University, Kunming, Yunnan, 650091, China.

^2^ State Key Laboratory of Phytochemistry and Plant Resources in West China, Kunming Institute of Botany, Chinese Academy of Sciences, Kunming, Yunnan 650201, China.

^3^ Department of Respiratory and Critical Disease, Xi’an International Medical Center Hospital, Xi’an, Shaanxi, 710100, China.

^4^ Key Laboratory of Pu-erh Tea Science of Ministry of Education, Yunnan Research Center for Advanced Tea Processing, Yunnan Agricultural University, Kunming, China; No.452 FengYuan Street, Kunming, 650201, Yunnan, China.

**^*^ Corresponding author.**

^#^ Dan Luo and Jianping Xu contributed equally to this work.

**Supplemental materials**

The main composition of the normal diet used in this work was shown in below table1.

**Table S1. The main composition of normal diet.**

| Composition | Ingredients |
| --- | --- |
| Proteins | soybean meal, fish meal, beer yeast powder |
| Lipids | vegetable oil |
| Fibres | bran |
| Carbohydrates | corn, wheat |
| Vitamins | Vitamin A, Vitamin D, Vitamin E, Vitamin B1, Vitamin B2, Vitamin B6, pantothenic acid, etc. |
| Minerals | calcium hydrogen carbonate, Fe, Cu, Mn, Zn |
